# Supplementary material for: A rigid and healable polymer cross-linked by weak but abundant Zn(II)-carboxylate interactions
Source: Nat Commun. 2018 Jul 13;9:2725. doi: 10.1038/s41467-018-05285-3 (PMC6045665; doi:10.1038/s41467-018-05285-3)
Supplement: Supplementary file 1 — Supplementary Information [file 41467_2018_5285_MOESM1_ESM.pdf]

Supplementary Information for

A Rigid and Healable Polymer Cross-linked by Weak but Abundant Zn(II)-Carboxylate Interactions

Lai *et al.*

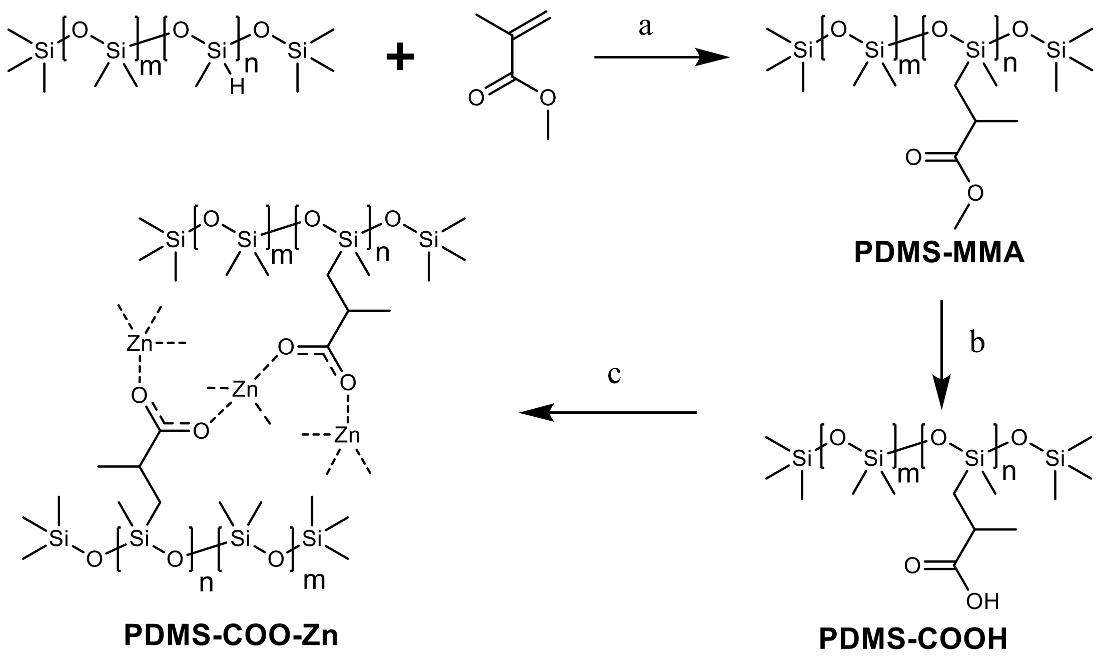

Supplementary Figure 1. Synthesis of the highly crosslinked **PDMS-COO-Zn** polymer. (**a**) Karstedt catalysis, toluene, 85 ℃, 10 h; (**b**) (1) LiOH, THF, H_2O, 85 ℃, 2 h, (2) HCl, THF, H_2O; (**c**) (1) $ZnCl_2$, Et_3N, EtOH, (2) DCM.

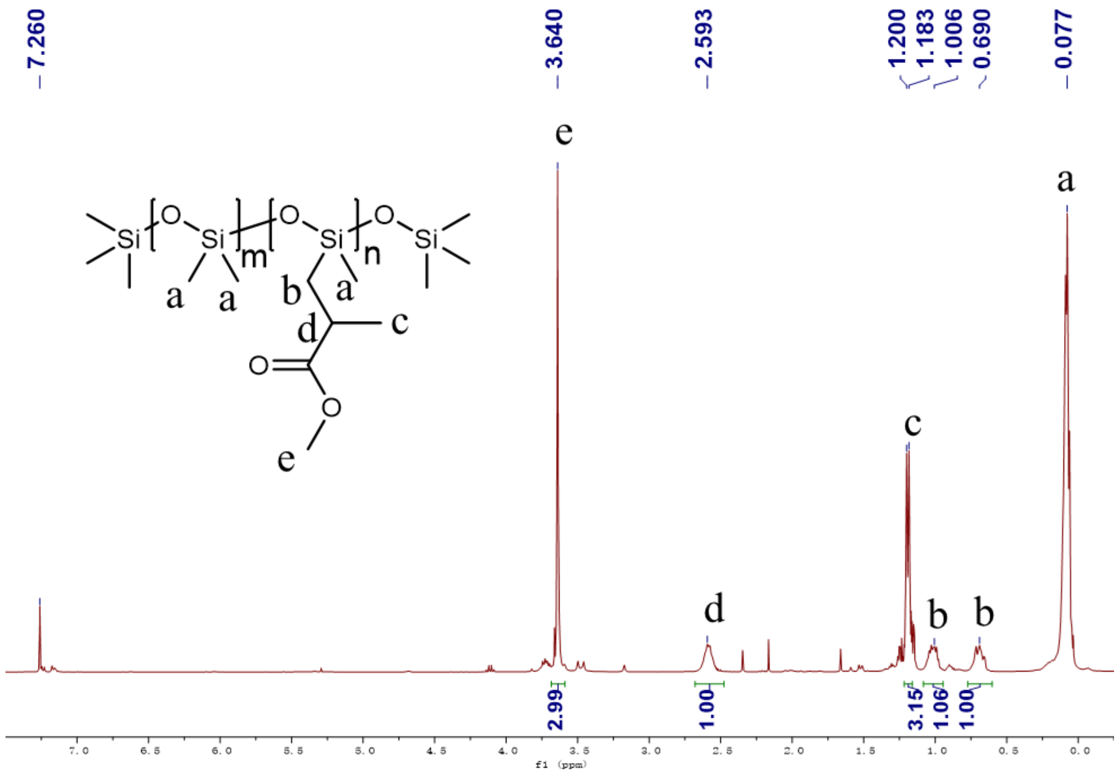

Supplementary Figure 2. ^{1}H NMR spectrum of **PDMS-MMA**.

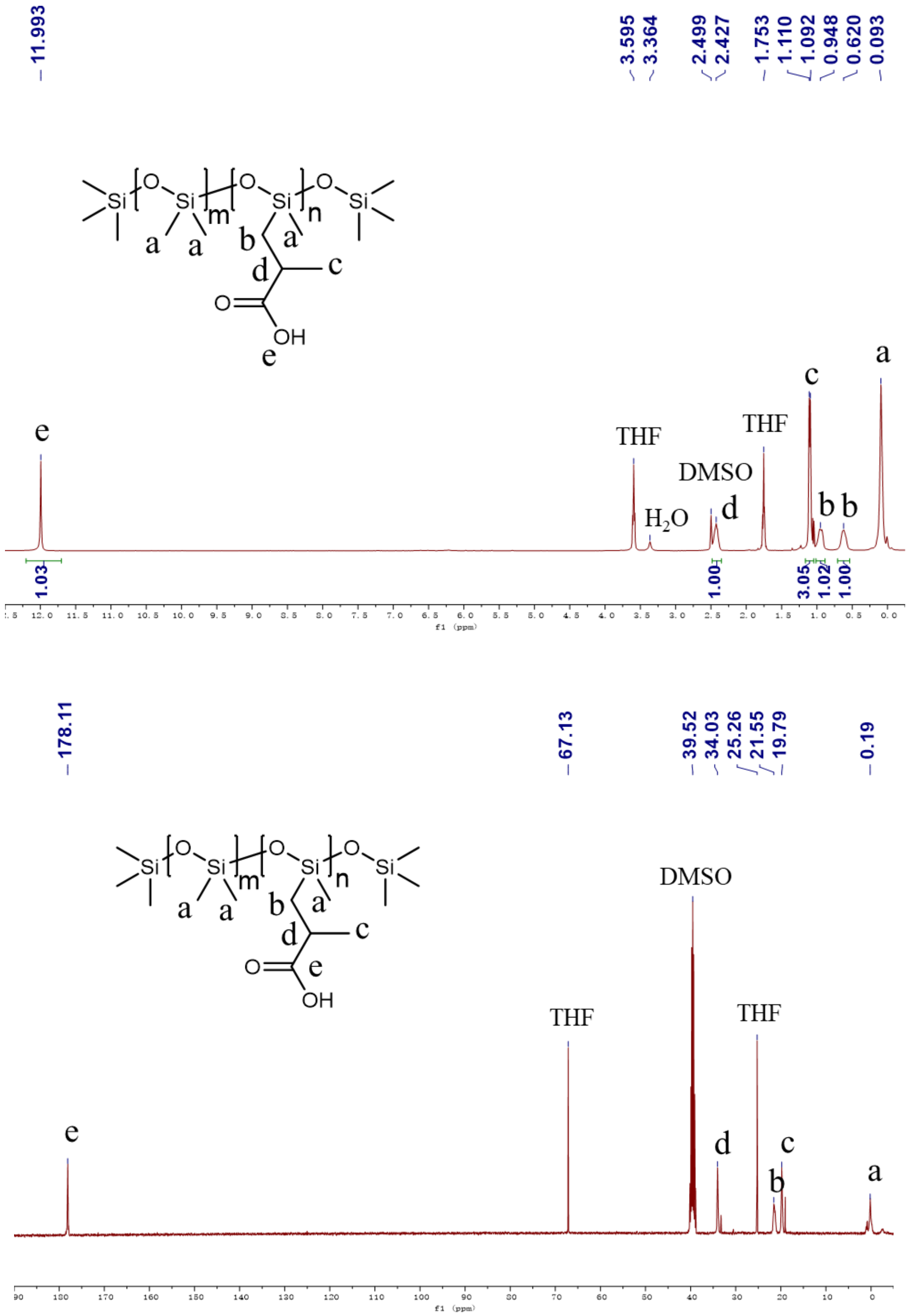

Supplementary Figure 3. ^{1}H NMR and ^{13}C NMR spectra of **PDMS-COOH.**

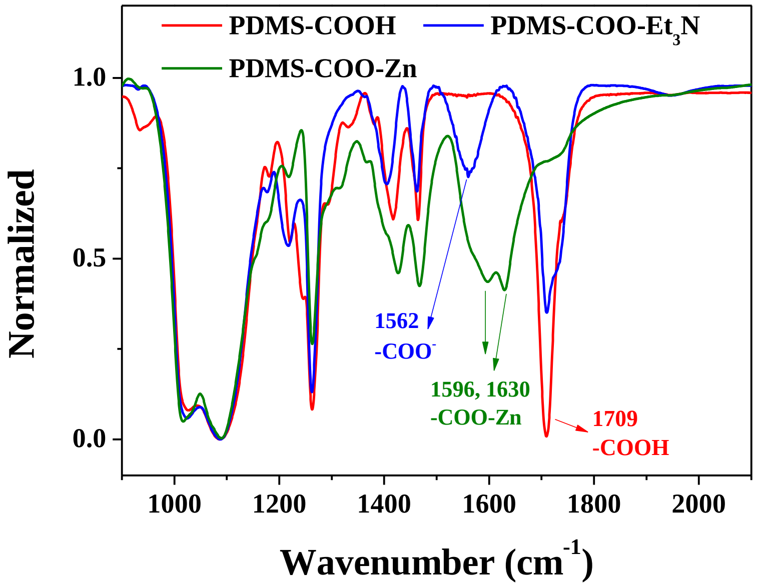

Supplementary Figure 4. FT-IR spectra of **PDMS-COOH, PDMS-COO-Et$_3$N** and **PDMS-COO-Zn.** The peak at 1596 cm^{-1} and 1630 cm^{-1} for **PDMS-COO-Zn** are significantly different to both carboxyl (1709 cm^{-1}) in **PDMS-COOH** and carboxylate (1562 cm^{-1}) in **PDMS-COO-Et$_3$N**, indicating the dominance of coordination bonds as the crosslinking interactions in **PDMS-COO-Zn**.

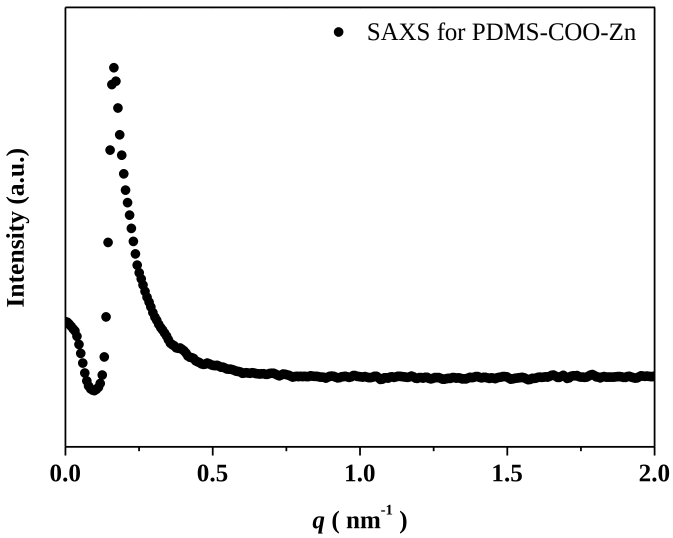

Supplementary Figure 5. The SAXS spectrum of the **PDMS-COO-Zn** polymer**.** No peak characteristic for cluster was observed in this spectrum, indicating that there is no obvious aggregations of Zn(II)-carboxylate complexes.

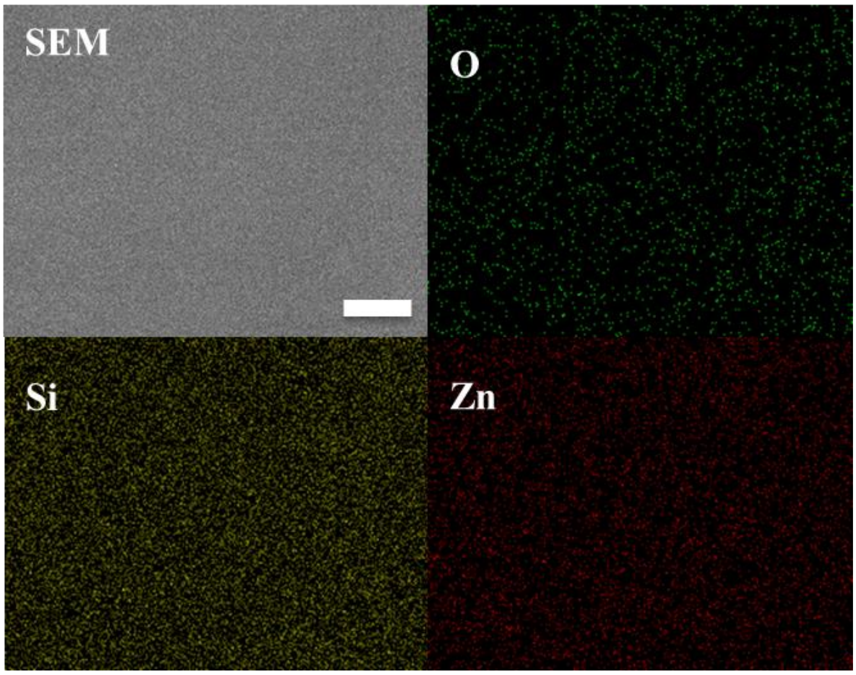

Supplementary Figure 6. The EDS (mapping mode) pictures of the **PDMS-COO-Zn** polymer shows that the homodisperse of Zn(II)-carboxylate complexes in the polymer**.** Scale bars, 1 μm.

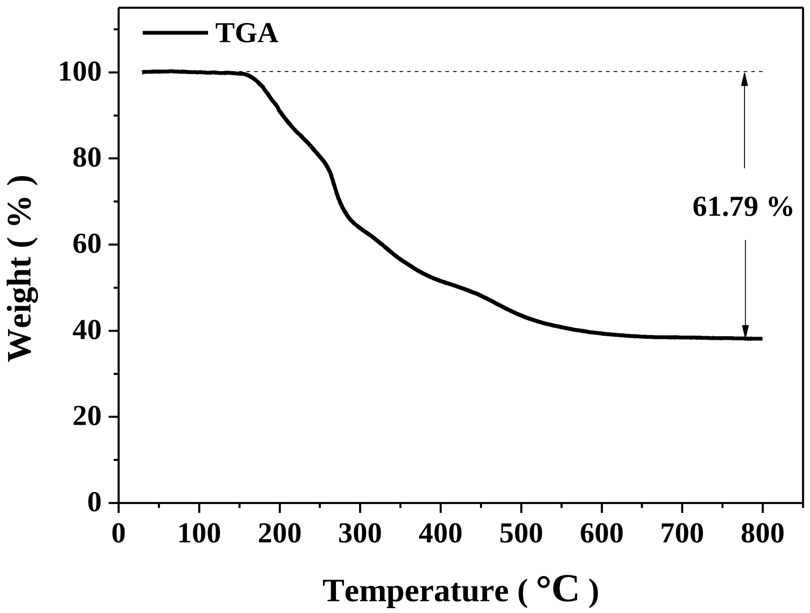

Supplementary Figure 7. The TGA curve of the **PDMS-COO-Zn** polymer.

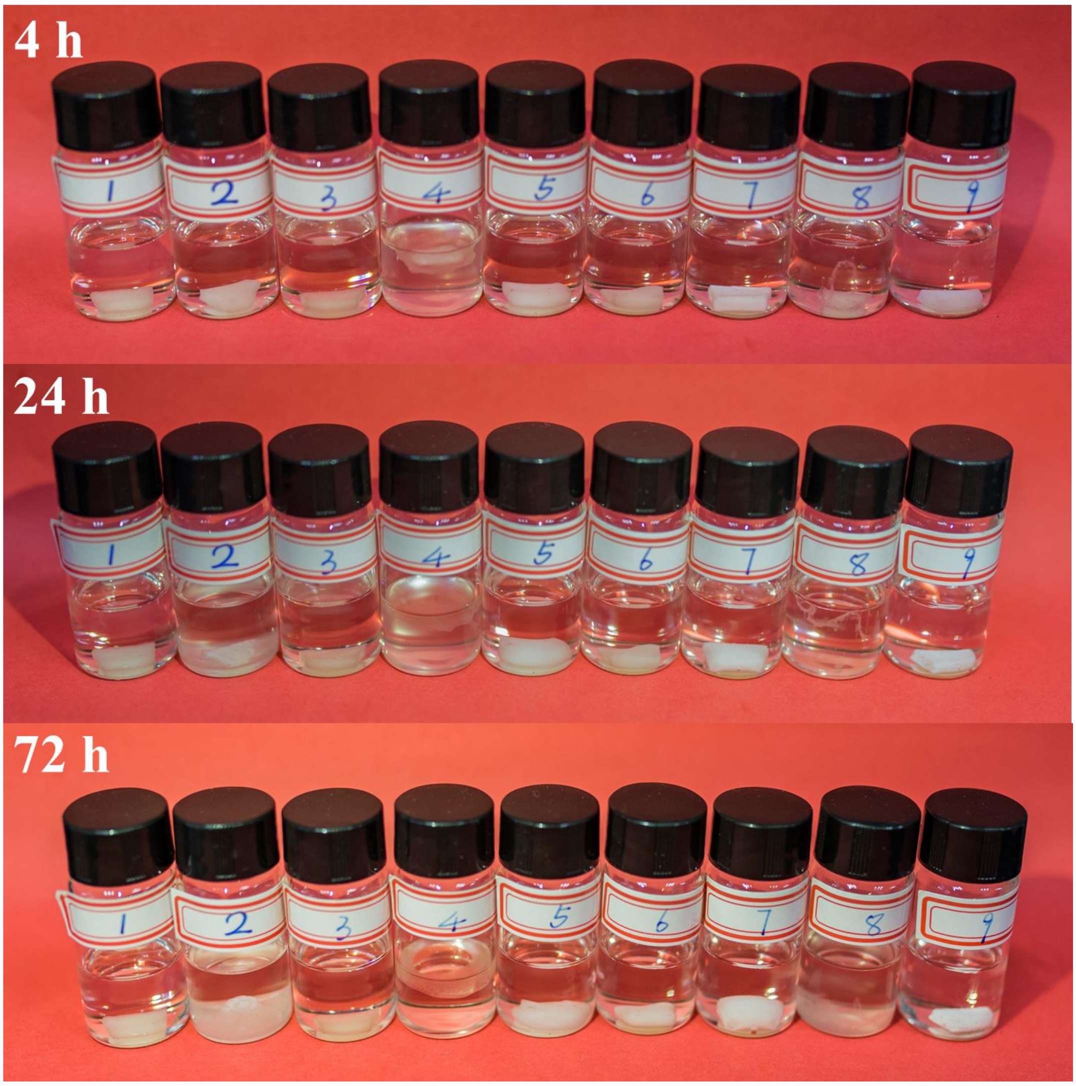

Supplementary Figure 8. The dissolution experiments for the **PDMS-COO-Zn** polymer. It shows that the polymer slightly swells in solvent 1, 3, 5, 6, 7 and 9, partially dissolve in solvent 2, 4 and 8. The corresponding solvents are as followed: 1. Hexane, 2. Ethanol, 3. Toluene, 4. Dichloromethane, 5. Tetrahydrofuran, 6. Ethyl acetate, 7. Acetonitrile, 8. Dimethyl formamide, 9. H_2O.

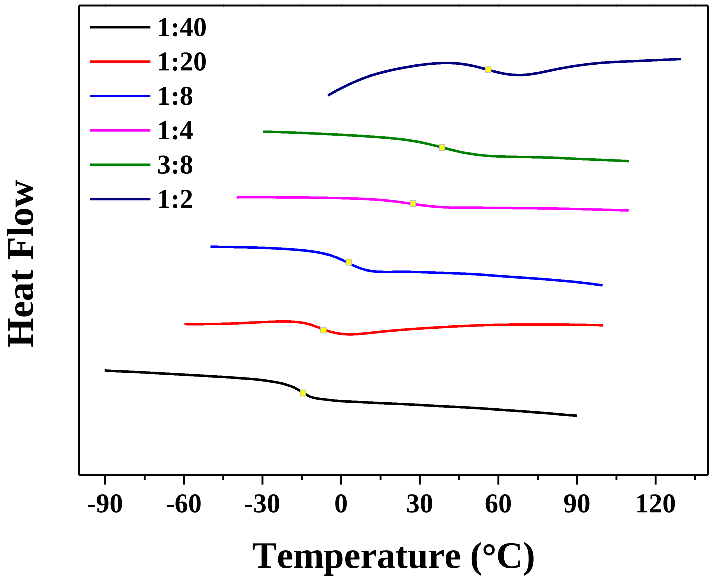

Supplementary Figure 9. DSC curves of the **PDMS-COO-Zn** polymer with different metal-to-ligand molar ratio, the yellow square shows the T_g of corresponding polymer, which revealed the T_g was downshift with decreasing the metal-to-ligand molar ratio and the T_g was about 55.7 ℃ when the metal-to-ligand molar ratio is 1:2.

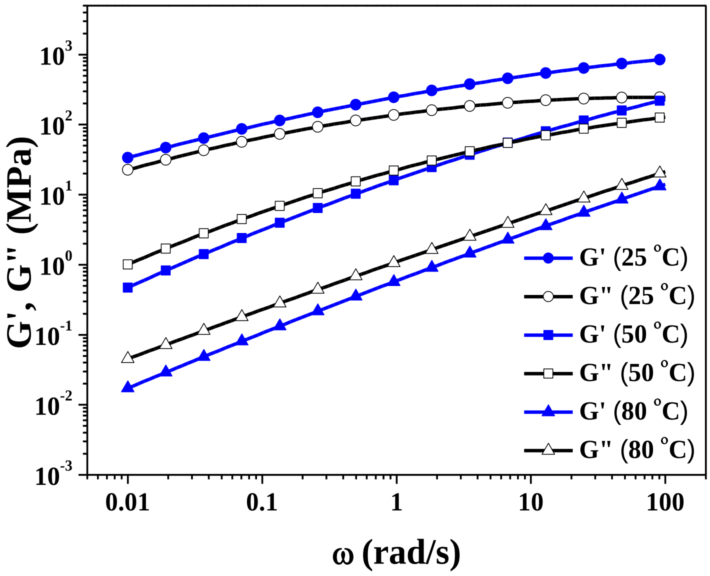

Supplementary Figure 10. Frequency sweep measurement of **PDMS-COO-Zn** at different temperature. The frequency sweep curves at 25 °C did not show a cross point between G' and G" even when the frequency was reduced to 10^{-2} rad/s.

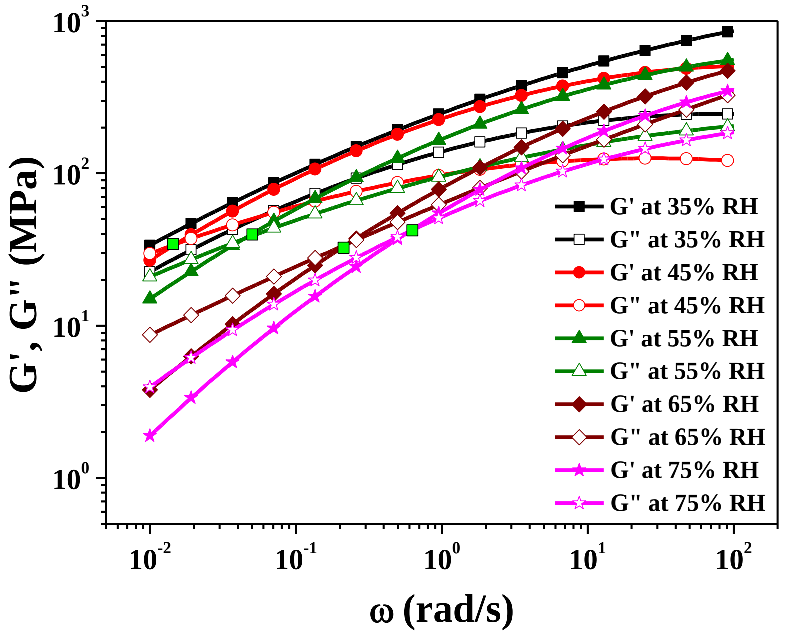

Supplementary Figure 11. Frequency sweep measurement of **PDMS-COO-Zn** at different relative humidity. The bright green squares represent the intersection of G' and G".

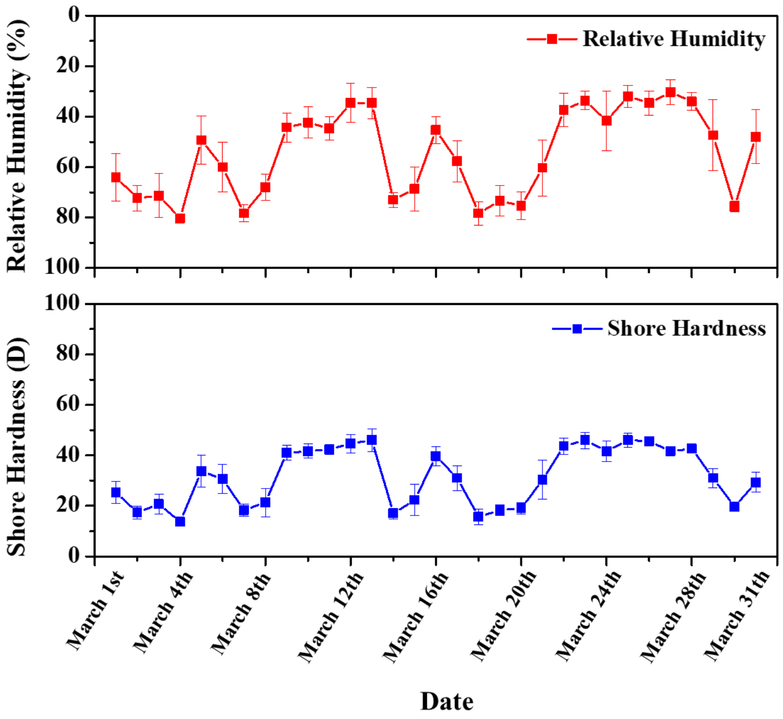

Supplementary Figure 12. The relative humidity and Shore hardness of sample for 30 days. The Shore-D hardness of the samples decreased with the increase of humidity but will be recovered when the humidity decreases. Error bars are the s.d. from triplicate measurements.

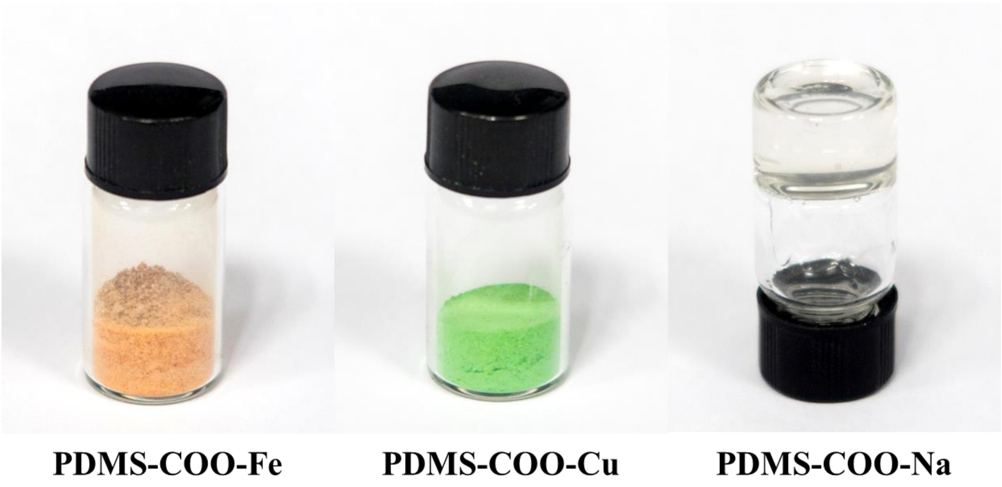

Supplementary Figure 13. Optical Photograph of **PDMS-COO-M** (**M = Fe^{3+}, Cu^{2+}, Na^{+}**), which shows that the PDMS-COO-Fe and PDMS-COO-Cu was unmoldable solid powder and PDMS-COO-Na was liquid-like gels.

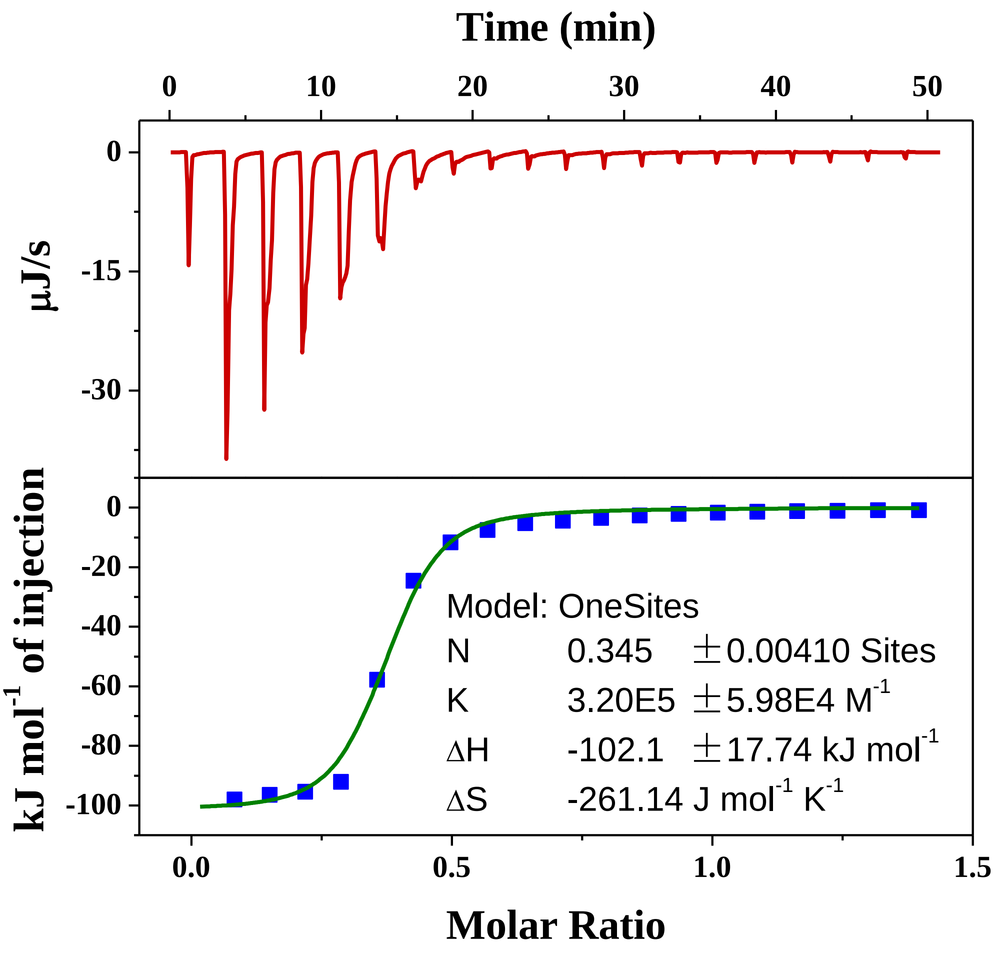

Supplementary Figure 14. The ITC titration data of the **PDMS-COO**$^{-}$ with $FeCl_3$ in anhydrous ethanol at 25 °C, which shows the K_a of **PDMS-COO-Fe** was about 8 times as much as **PDMS-COO-Zn**.

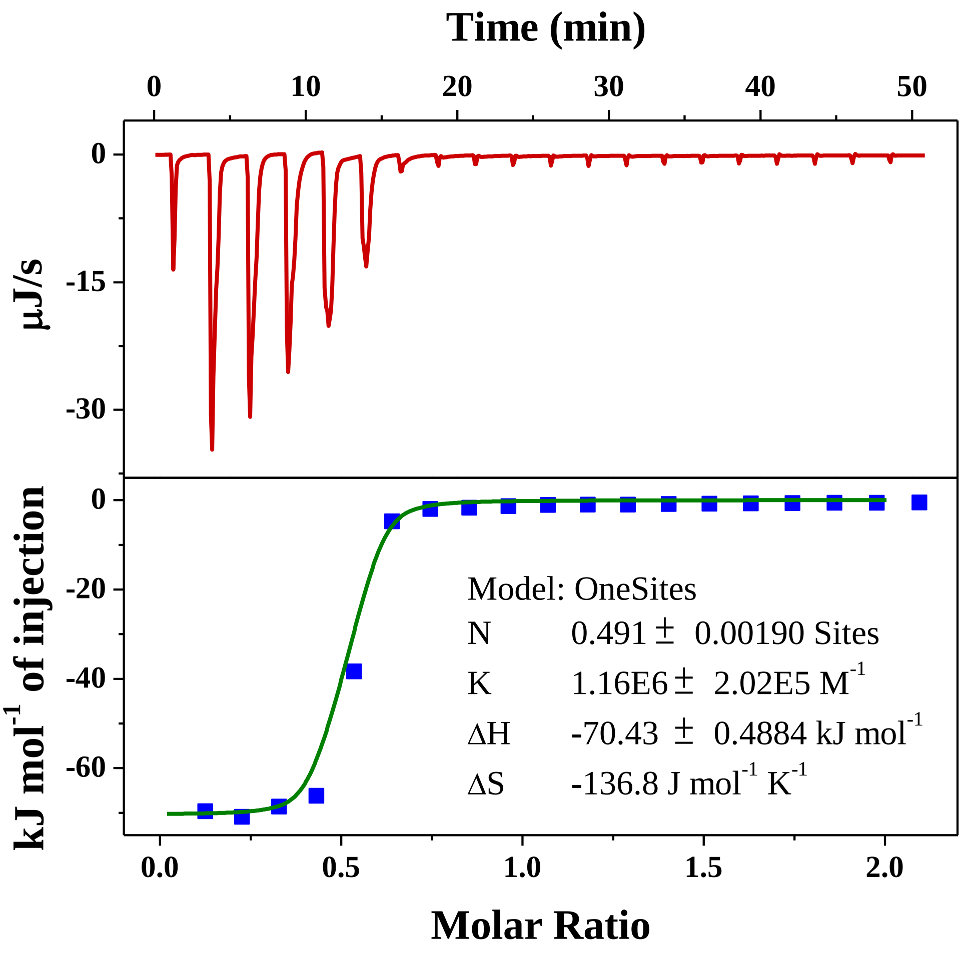

Supplementary Figure 15. The ITC titration data of the **PDMS-COO$^-$** with $CuCl_2$ in anhydrous ethanol at 25 °C, which shows the K_a of **PDMS-COO-Cu** was about 28 times as much as **PDMS-COO-Zn**.

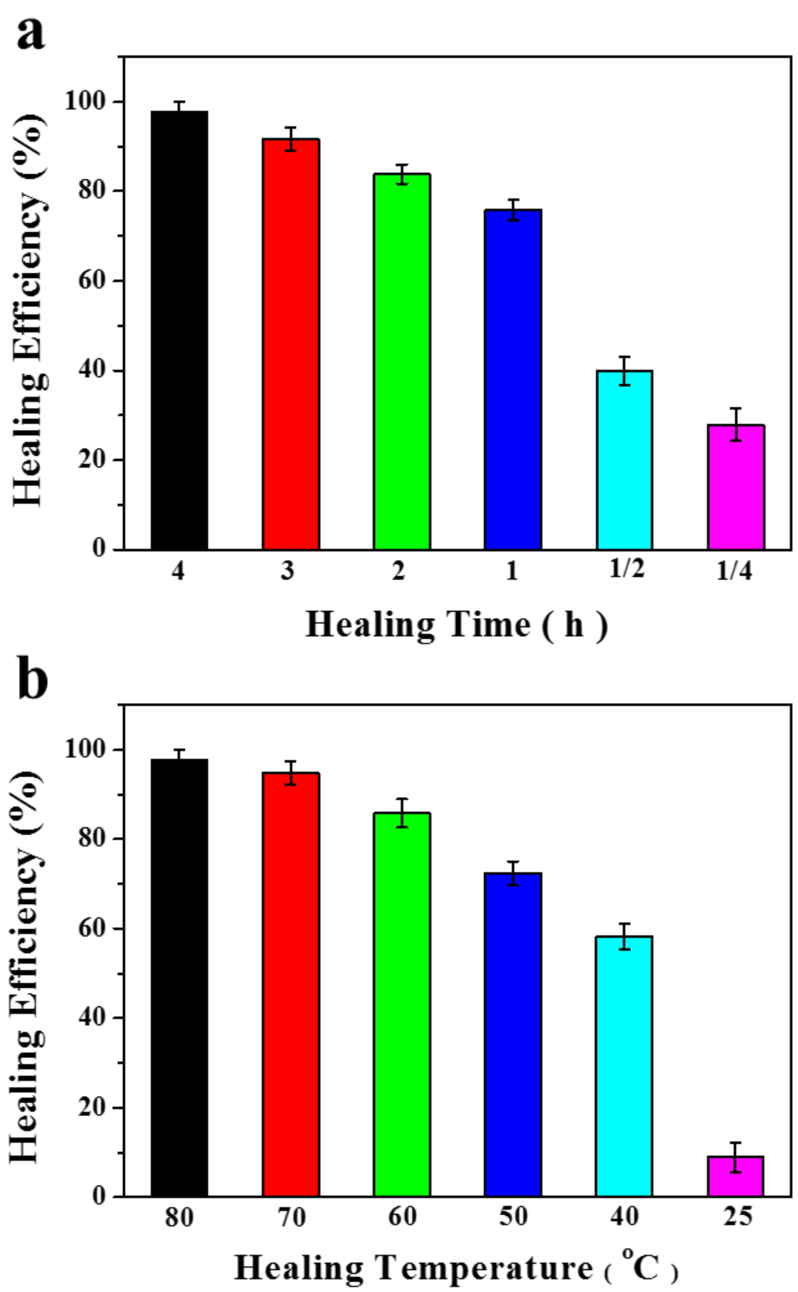

Supplementary Figure 16. The healing efficiencies of **PDMS-COO-Zn** under different conditions. (**a**) Healing at 80 ℃ with different length of time. (**b**) Healing for 4h at different temperature. Error bars are the s.d. from quadruplicate measurements. The self-healing efficiency is calculated as follows:

$$Healing\ \ efficiency = \frac{Maximal\ \ strength_{healed}}{Maximal\ \ strength_{original}} \times 100\%$$

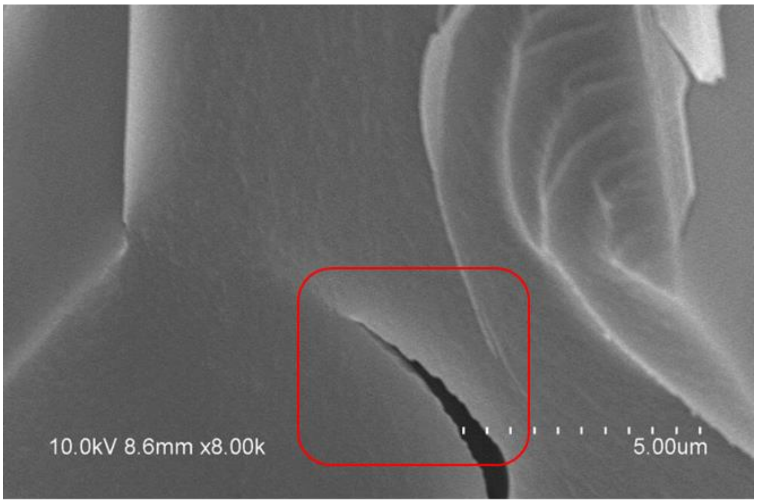

Cracks formation

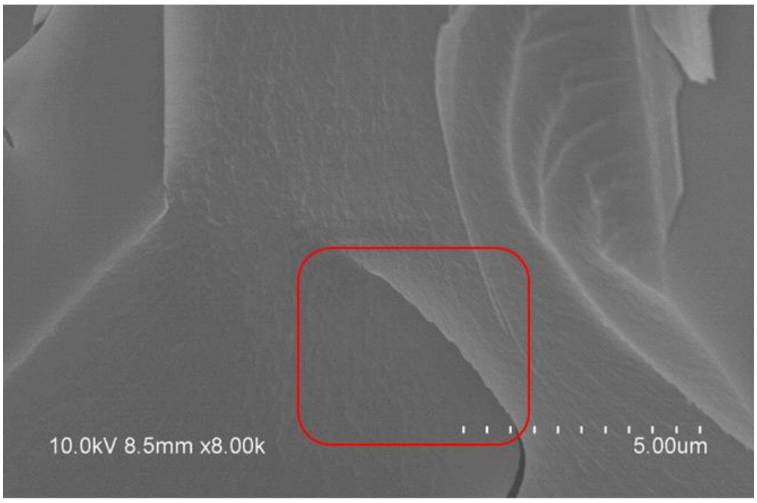

Cracks healing after 300 s

Supplementary Figure 17. Self-healing process observed in SEM microscope under the successive scan of 300 s.

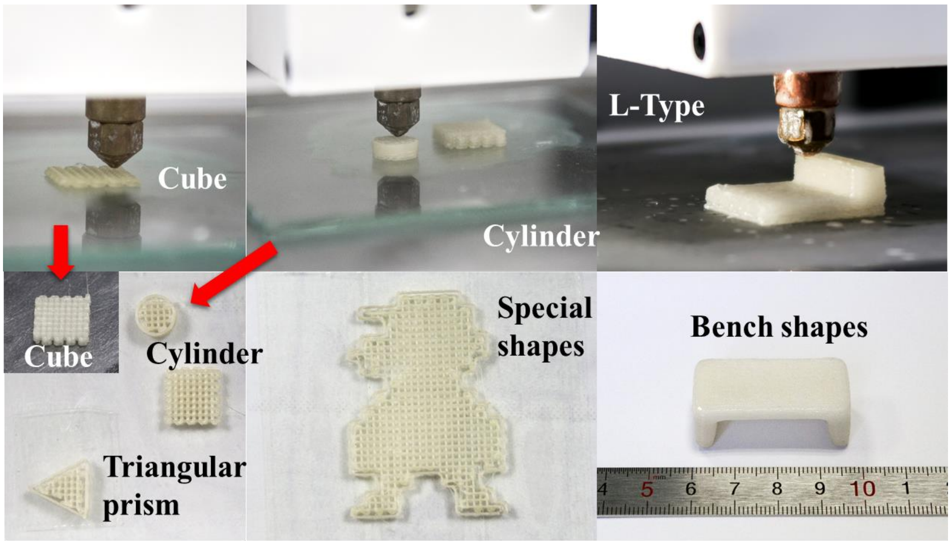

Supplementary Figure 18. Various shapes obtained from 3D printing with **PDMS-COO-Zn** polymer.

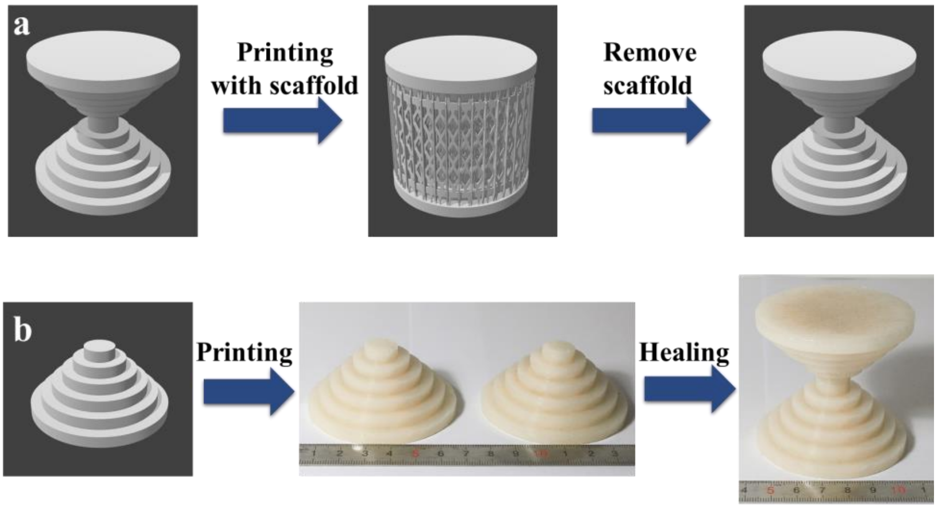

Supplementary Figure 19. The comparation of traditional 3D printing method (**a**) and self-healing 3D printing method (**b**). If we use 3D printer to make such object, we have to print many scaffolds to support the top weight. The scaffolds have to be removed after printing to afford the desired shape (**Supplementary Figure 19a**). Such a process is tedious and wasteful. However, in our procedure, we only need to print two pedestals and then assemble them into the desired shape through thermal healing (**Supplementary Figure 19b**). As the pedestal is large in the bottom and small in the top, no scaffold is needed. Therefore, we can combine the advantages of modern 3D printing processes and brick-and-mortar operations with self-healing technology.

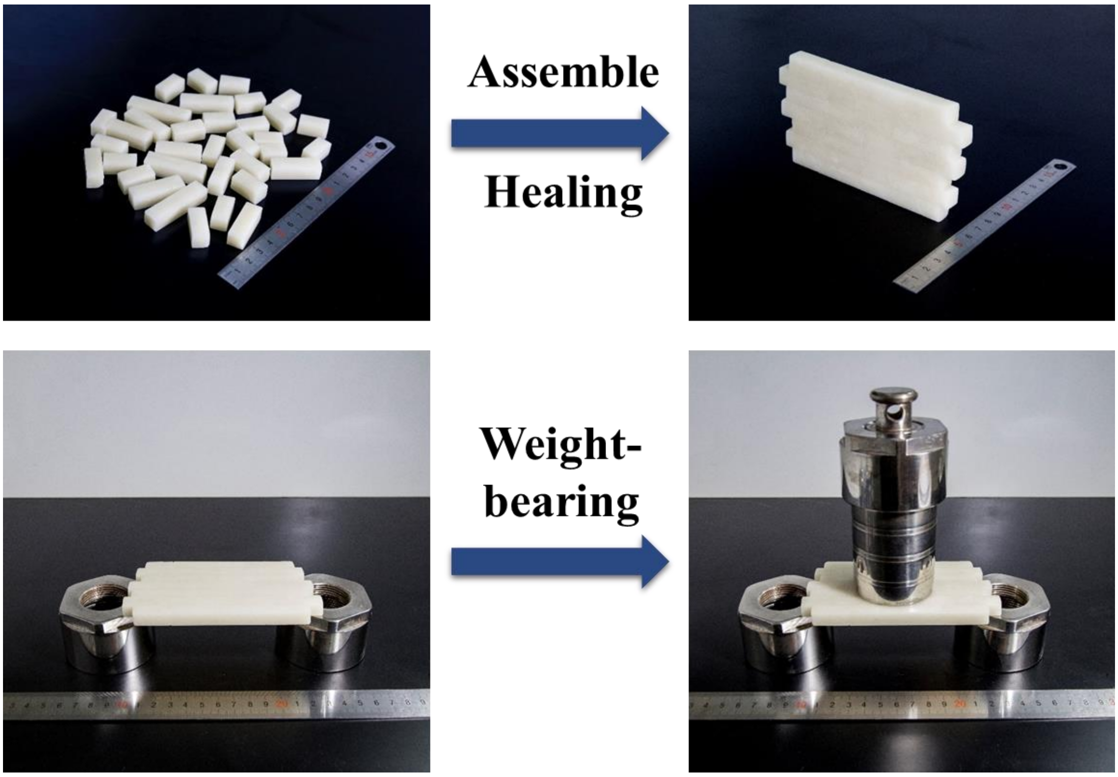

Supplementary Figure 20. Weight-bearing test of the healed integrated wall.

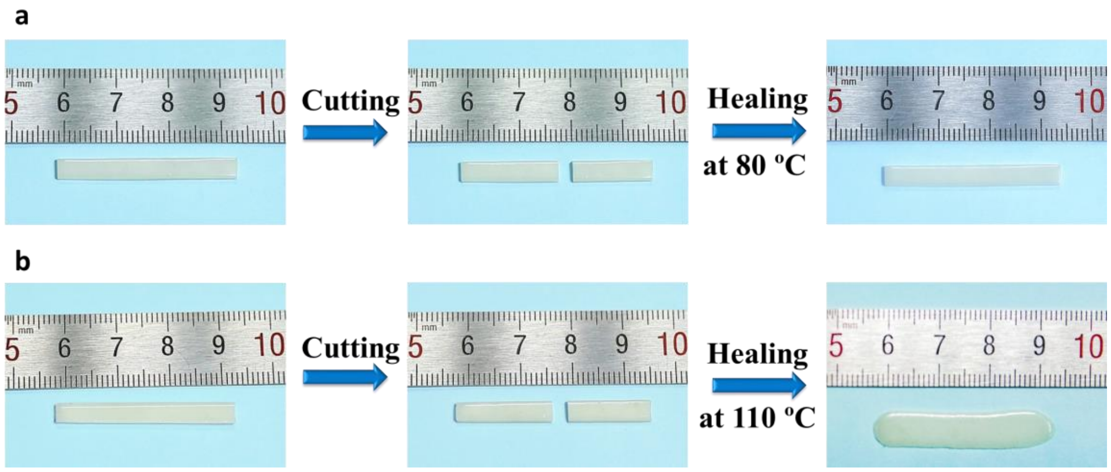

Supplementary Figure 21. The self-healing process carried out under different temperature for 4 h and the corresponding shape deformation. (**a**) Healing at 80 °C, (**b**) Healing at 110 °C. The sample healed at 80 ℃ do not change shape but the samples healed at 110 ℃ exhibit obvious shape deformation.

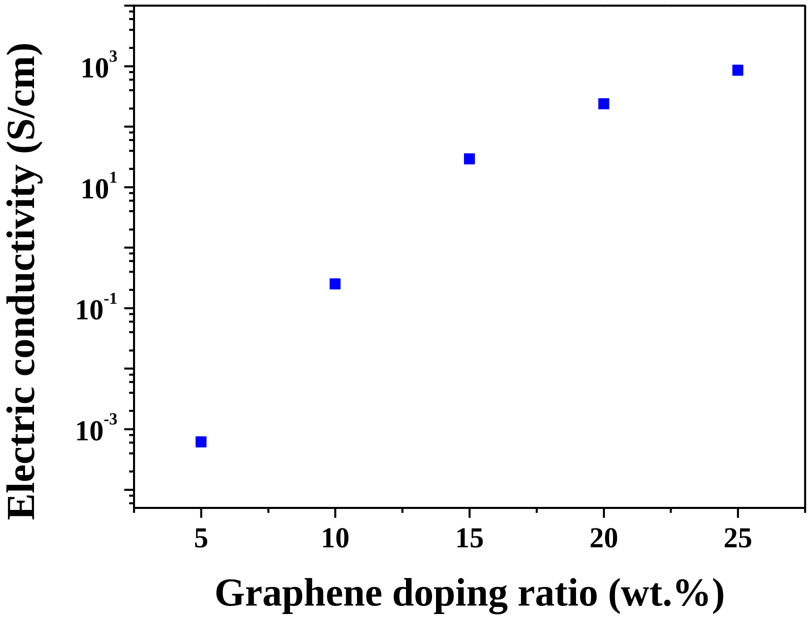

Supplementary Figure 22. Electric conductivity of **PDMS-COO-Zn**/Graphene composites as a function of graphene loading.

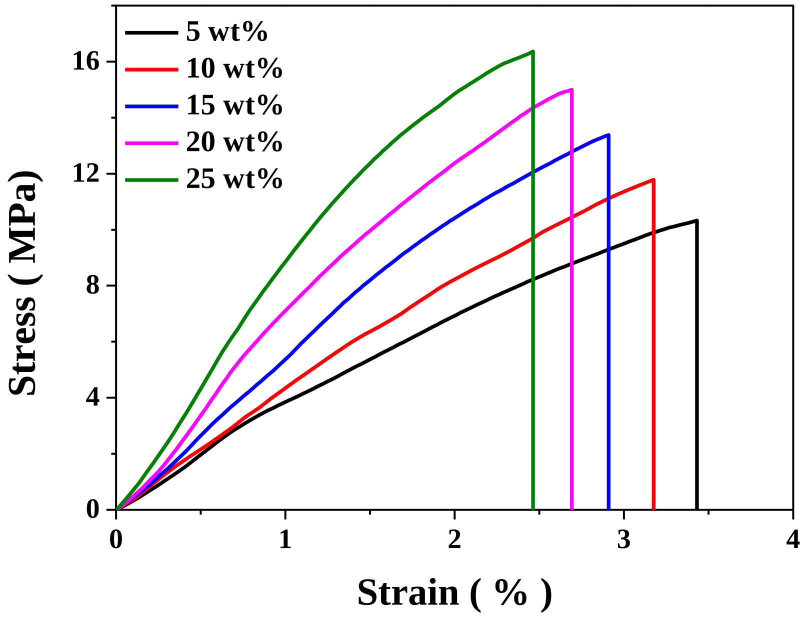

Supplementary Figure 23. The Three-point flexural stress-strain curves of the conducting composites with different doping ratio.

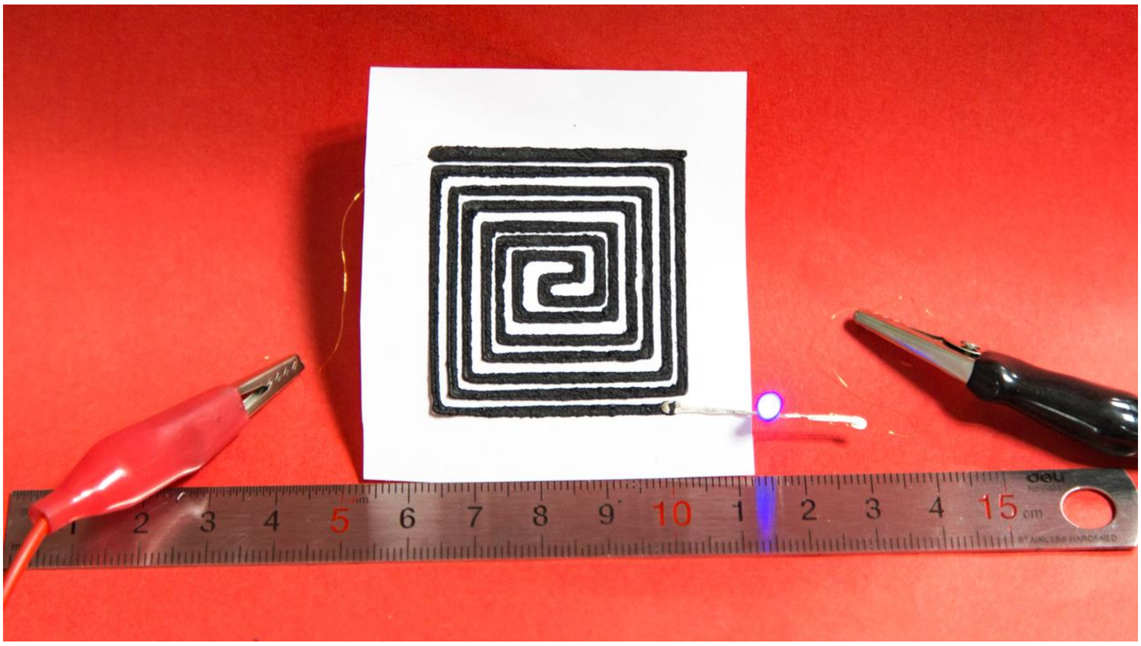

Supplementary Figure 24. The 3D printed electrical circuit can power the LED device.

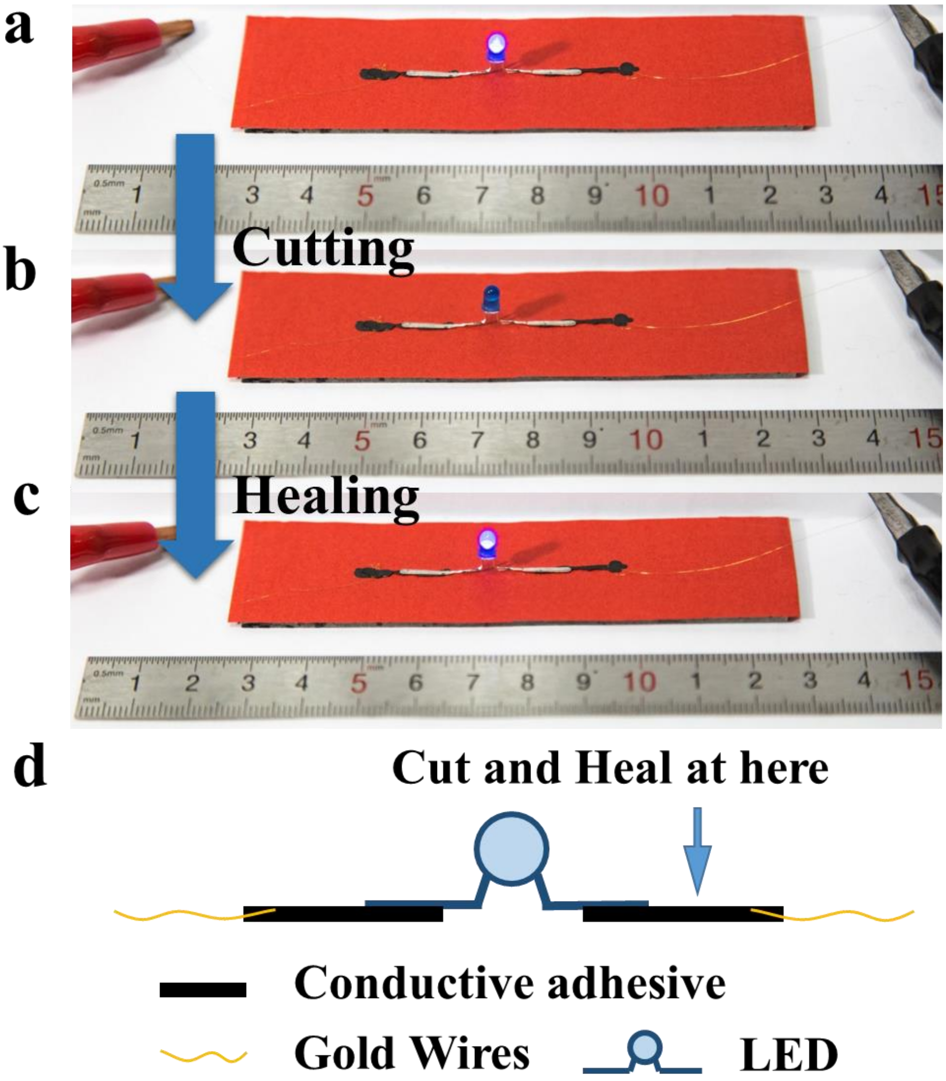

Supplementary Figure 25. The healable properties of the 3D printable electrical circuit.

Supplementary Table 1. Results of the isothermal titration calorimetry (ITC) of the **PDMS-COO**$^-$ (0.6 mM, in cell) with $ZnCl_2$ (6 mM, in syringe) in anhydrous ethanol at 25 ℃.

Model	Variable	Value
Onesites	Ka (M^{-1})	$4.10\times10^4\pm2.59\times10^3$
	N	0.605 ± 0.004
	ΔH (kJ mol^{-1})	-44.69 ± 0.42
	ΔS (J mol^{-1} K^{-1})	-72.40

Supplementary Table 2. The key mechanical properties of **PDMS-COO-Zn** under the flexural testing mode.

	**PDMS-COO-Zn** (mean ± s.d., n=4)
Young's modulus (MPa)	478.13 ± 20.25
Maximal strength (MPa)	9.146 ± 0.45
Breaking strain (%)	3.39 ± 0.39

Supplementary Table 3. Metal-to-ligand molar ratio dependence of T_g and Young's modulus of **PDMS-COO-Zn**

Metal to Ligand Molar Ratio	T_g (°C)	Young's Modulus (MPa)
1:2	55.7	478.13
3:8	38.4	285.27
1:4	27.2	112.15
1:8	2.5	12.96
1:20	-6.8	1.58
1:40	-14.6	0.25

Supplementary Table 4. The healing efficiency of **PDMS-COO-Zn** with various healing time and healing temperature.

Healing time (80 ℃, h)	Maximal strength (MPa, mean ± s.d., n=4)	Healing Efficiency (%, mean ± s.d., n=4)
Original	9.146	-
4	8.923 ± 0.215	97.56 ± 2.35
3	8.386 ± 0.235	91.69 ± 2.57
2	7.667 ± 0.198	83.83 ± 2.16
1	6.928 ± 0.215	75.75 ± 2.35
1/2	3.639 ± 0.286	39.79 ± 3.13
1/4	2.555 ± 0.324	27.94 ± 3.54
**Healing temperature (4 h, ℃)**	**Maximal strength (MPa, mean ± s.d., n=4)**	**Healing Efficiency (%, mean ± s.d., n=4)**
Original	9.146	-
80	8.923 ± 0.215	97.56 ± 2.35
70	8.677 ± 0.229	94.87 ± 2.51
60	7.849 ±0.210	85.82 ±2.29
50	6.621 ± 0.249	72.39 ± 2.72
40	5.335 ± 0.262	58.33 ± 2.87
25	0.822 ± 0.299	8.99 ± 3.27
