# Supplementary material for: A rigid and healable polymer cross-linked by weak but abundant Zn(II)-carboxylate interactions
Source: Nat Commun. 2018 Jul 13;9:2725. doi: 10.1038/s41467-018-05285-3 (PMC6045665; doi:10.1038/s41467-018-05285-3)
Supplement: Supplementary file 3 — Description of Additional Supplementary Files [file 41467_2018_5285_MOESM3_ESM.pdf]

Description of Additional Supplementary Files

File Name: Supplementary Movie 1
Description: Ceramic-like properties of **PDMS-COO-Zn**.

File Name: Supplementary Movie 2
Description: Variable stiffness properties of **PDMS-COO-Zn**.

File Name: Supplementary Movie 3
Description: **PDMS-COO-Zn** used as the orthopedic immobilization.

File Name: Supplementary Movie 4
Description: 3D printing with **PDMS-COO-Zn** polymer.

File Name: Supplementary Movie 5
Description: Build a house by 3D printing and thermal-healing.

File Name: Supplementary Movie 6
Description: 3D printing of conductive circuit.
